# SARS-CoV-2 Delta VOC in a Paucisymptomatic Dog, Italy

**DOI:** 10.3390/pathogens11050514

**Published:** 2022-04-26

**Authors:** Ilaria Pascucci, Marta Paniccià, Monica Giammarioli, Massimo Biagetti, Anna Duranti, Pamela Campomori, Valerio Smilari, Massimo Ancora, Silvia Scialabba, Barbara Secondini, Cesare Cammà, Alessio Lorusso

**Affiliations:** 1 Istituto Zooprofilattico Sperimentale dell’Umbria e delle Marche—Togo Rosati, 06126 Perugia, Italy; m.paniccia@izsum.it (M.P.); m.giammarioli@izsum.it (M.G.); m.biagetti@izsum.it (M.B.); a.duranti@izsum.it (A.D.); 2 Servizio Veterinario Sanità Animale Area Vasta 1-ASUR Marche, 61121 Pesaro, Italy; pamela.campomori@sanita.marche.it (P.C.); valerio.smilari@sanita.marche.it (V.S.); 3 Istituto Zooprofilattico Sperimentale dell’Abruzzo e Molise, 64100 Teramo, Italy; m.ancora@izs.it (M.A.); s.scialabba@izs.it (S.S.); b.secondini@izs.it (B.S.); c.camma@izs.it (C.C.); a.lorusso@izs.it (A.L.)

**Keywords:** SARS-CoV-2, Italy, Delta VOC, dog

## Abstract

Since the very beginning of the COVID-19 pandemic, SARS-CoV-2 detection has been described in several animal species. A total of 625 outbreaks in animals have been reported globally, affecting 17 species in 32 countries and the human source of infection has been recognized including pet owners, zookeepers, and farmers. In this report, we describe the case of a paucisymptomatic dog in Italy infected with SARS-CoV-2 from a household with three confirmed human cases of COVID-19 living in Pesaro (Marche region, Italy). The dog showed high viral RNA titers in the nasal and oropharyngeal swabs. In the nasal swab, SARS-CoV-2 RNA lasted for a least a week. By sequencing, the strain was assigned to the AY.23 lineage (PANGO), one of the sub-lineages of the major SARS-CoV-2 Delta variant of concern (VOC). Although we did not process the swabs of the three human cases, we strongly suspect a human origin for the dog infection. In this regard, AY.23 sequences, although never released thus far in the Marche region, were detected in the neighboring regions. Our findings highlight once more the need for a One Health approach for SARS-CoV-2 surveillance, management, and control, thus preventing viral spillover from animals to humans.

## 1. Introduction

The zoonotic severe acute respiratory syndrome coronavirus 2 (SARS-CoV-2, Family *Coronaviridae*, genus *Betacoronavirus*; subgenus *Sarbecovirus*; species *severe acute respiratory syndrome-related coronavirus*) is responsible for the ongoing pandemic of coronavirus infectious disease 2019 (COVID-19) that, up to 28 February of 2022, has caused 434,154,739 confirmed cases of COVID-19, including 5,944,342 deaths [1]. Horseshoe bats (genus *Rhinolophus*) are recognized as the natural reservoirs of SARS-related CoVs and the likely origin of SARS-CoV-1 and SARS-CoV-2 [2,3]. Coronaviruses are characterized by exceptional genetic plasticity. Indeed, besides the heterologous and homologous recombination capabilities, the viral replicase (an RNA dependent RNA polymerase) does not possess a good proofreading activity; therefore, the incorporation of wrong nucleotides at each replication cycle and the consequent accumulation of mutations in the viral genome lead to a progressive differentiation of the viral progeny from the parental strain. This mechanism led to the emergence of several SARS-CoV-2 variants, characterized by different biological properties with respect to the SARS-CoV-2 prototype strain. Mutations occur in the ORF coding for the spike (S) protein—the outermost protein of the virion and key in entering the host cell by binding to the angiotensin-converting enzyme 2 (ACE2)—may affect infectivity, tropism, immune system evasion, and host range. Some variants have been recognized as variants of concern (VOC), and, as of 10 February 2022, five VOCs including Alpha or PANGO lineage B.1.1.7 (first described in the UK), Beta or PANGO lineage B.1.351 (initially identified in South Africa), Gamma or PANGO lineage P.1 (first described in Brazil), Delta or PANGO lineage B.1.617.2 (initially detected in India), and Omicron or lineage B.1.1.529 (identified for the first time in Botswana) have been described [4]. Since the beginning of the pandemic, SARS-CoV-2 detection has been described in several animal species [5,6]. A total of 625 outbreaks in animals have been reported globally, affecting 17 species in 32 countries [5], and the human source of infection has been recognized including pet owners, zookeepers, and farmers [7]. In this framework, due to their close contact with humans, pets have received the most attention, with a total number of 102 SARS-CoV-2-RNA-positive domestic cats and 86 dogs [8]. SARS-CoV-2 infection has been described recently in Italy in two asymptomatic dogs living in a COVID-19-affected family [9]. Genome analysis demonstrated that dogs and their owner were infected with the same strain belonging to the B.1.177 lineage, responsible for the surge of COVID-19 cases in humans in summer–autumn of 2020 in Italy [10,11]. The present paper describes the case of a paucisymptomatic dog from Italy infected with the SARS-CoV-2 Delta VOC.

## 2. Materials and Methods

On 20 December 2021, a 12-year-old mixed-breed female dog from a household with three confirmed human cases of COVID-19 living in Pesaro (Marche region, Italy) developed upper respiratory tract symptoms with frequent sneezing. The dog presented clinical signs six days after SARS-CoV-2 detection of its related human cases. Samples collection of feces, nasal, and oropharyngeal swabs were taken immediately from the dog. Total RNA extraction was carried out onto an automatic extractor by using the MagMax viral/pathogen II Nucleic Acid Isolation Kit (Applied Biosystems ™ Waltham, Massachusetts, USA). Purified RNA was tested by means of TaqPath COVID 19 CE-IVD RT-PCR Kit (Applied Biosystems™). According to Istituto Superiore di Sanità (ISS) guidelines, specimens from the dog were collected two times on 27 December 2021 and on 3 January 2022.

### Library Preparation COVIDSeq Test (Illumina) Automated on the Hamilton Microlab STAR Liquid Handling System

Total RNA extracted from the nasal swab on 27 December 2021 was selected for whole-genome sequencing (WGS) by using the COVIDSeq Test (Illumina Inc., San Diego, CA, USA), along with the daily routine samples. This library preparation method was automated in-house and validated for the Hamilton Microlab STAR Liquid Handling System (Hamilton Robotics, Reno, NV, USA) at the National Reference Center for Whole-Genome Sequencing of microbial pathogens: database and bioinformatic analysis (GENPAT) of IZS-Teramo. Deep sequencing was performed on the NextSeq 500 platform (Illumina Inc., San Diego, CA, USA) using the NextSeq 500/550 Mid Output Reagent Cartridge v2, 300 cycles, and standard 150 bp paired-end reads. After quality control and trimming of the reads by FastQC and Trimmomatic, mapping to the Wuhan-Hu-1 reference genome (Acc no NC_045512) was performed by the BWA tool [12], and the consensus sequence was obtained using iVar (v1.3.1) (intrahost variant analysis of replicates; github.com/andersen-lab/ivar). All analysis steps were automatically performed at the end of the sequencing run on the GENPAT platform at IZS-Teramo, as described [13]. The identification of the occurring SARS-CoV-2 lineage was confirmed by using the PANGO tool via the web (https://pangolin.cog-uk.io/ (accessed on 14 February 2022)).

## 3. Results

**Figure 1:**
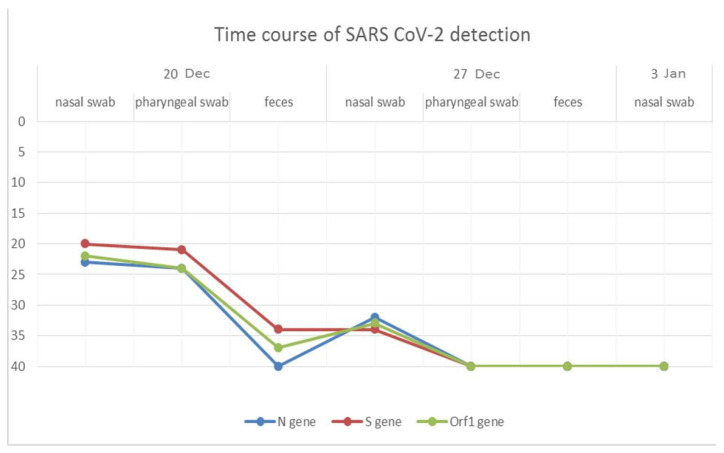
Time course of SARS-CoV-2 detection in dog samples. Y axis, CT values; Dec, December; Jan, January.

The infected dog showed high RNA titers in the oropharyngeal and nasal swabs, and SARS-CoV-2 RNA was determined with threshold cycle (CT) values of 24 for the N gene (CT N), 21 for the S gene (CT S), and 24 for the ORF1ab gene (CT ORF1ab), and 23 CT N, 20 CT S, and 22 CT ORF1ab in the oropharyngeal and nasal swab, respectively, collected on 20 December 2021. Conversely, the fecal sample tested positive only for two targets with higher CT values (CT S 34 and CT ORF1ab 37). Out of three specimens collected on 27 December 2021, when complete remission of symptoms was observed, only the nasal swab tested positive for SARS-CoV-2 RNA, with 32 CT N, 34 CT S, and 33 CT ORF1ab. Nasal swab collected on 3 January 2022 tested negative for SARS-CoV-2 RNA for all three targets (Figure 1). Serum samples were not collected.

### The Occurring Strain Belonged to the AY.23 PANGO Lineage (Delta VOC)

The settled automated method adopted in this study reproduced the manual processing steps to improve automated liquid handling. This allowed us to have a fully automated pipeline with high-throughput systems in terms of both a time-saving procedure and reproducibility. Importantly, it also provided the elimination of potential amplicon contamination thus assuring a safer and more efficient laboratory workflow. The sequencing run produced a total number of 5,080,112 raw reads, with an average quality score of 33.2 per sample. Out of them, 835.638 reads were mapped on the Wuhan-Hu-1 reference genome with average depth coverage of 4.013X. The consensus sequence obtained belonged to AY.23 PANGO lineage (last accessed 10 February 2021), a sub-lineage of the major Delta VOC. Sequence (hCoV-19/dog/Italy/ABR-IZSGC-319425/2021) has been released on GISAID under accession number EPI_ISL_9906009. No AY.23 sequences were so far released from the Marche region. Nevertheless, according to GISAID, AY.23 sequences were released from the neighboring Abruzzo, Umbria, and Emilia–Romagna regions.

## 4. Discussion and Conclusions

In the present study, the infection of a dog with one of the sub-lineage (AY.23) of the Delta VOC was described. COVID-19 infection of this dog was characterized by higher viral RNA loads, compared with similar reports described in Italy [9] and Spain [14]. In this latter case, the dog was also infected with a Delta VOC strain and displayed mild digestive and respiratory clinical signs but with a lower viral load in the oropharyngeal swab at the first sampling. In our case, despite the presence of mild clinical signs, viral loads in the nasal and oropharyngeal swabs were higher and long-lasting, as they persisted for at least one week in the nasal swab. As already demonstrated from previous studies, close contact with the infected owners was likely responsible for the dog infection. In this regard, the main pitfall of the report is the absence of the genome analysis of SARS-CoV-2 strains infecting the dog’s related human cases, which hampered a proper and conclusive epidemiological analysis. Furthermore, serum samples were not collected from the infected dog; thus, we cannot demonstrate seroconversion. Notably, a robust conclusion upon characteristics of AY.23 infection in dogs cannot be drawn. In this regard, timely surveillance of pets at risk of infection should be implemented. Overall, the evolutionary characteristics of SARS-CoV-2 may provide a long-lasting emergence of additional new variants also from animals with different biological properties, including higher transmissibility and virulence, thus requiring the maintenance of a high warning system for SARS-CoV-2 infections in animals. 

Our findings highlight once more the need for a One Health approach for SARS-CoV-2 surveillance, management, and control, thus preventing spillover from animals to humans [15,16].

## Data Availability

The data presented in this study are available in the present article.
